# The role of Mediterranean diet adherence, smoking and their interactions in epigenetic age acceleration: A cross-sectional analysis of the Airwave cohort

**DOI:** 10.1016/j.jnha.2026.100964

**Published:** 2026-08-28

**Authors:** Azaan Zaki, Chung-Ho E. Lau, Rebeca Eriksen, Gary Frost, Ian Mudway, Oliver Robinson

**Affiliations:** a MRC Centre for Environment and Health, Environmental Research Group, Imperial College London, London, United Kingdom; b MRC Centre for Environment and Health, Department of Epidemiology and Biostatistics, Imperial College London, United Kingdom; c Ageing Epidemiology (AGE) Research Unit, School of Public Health, Imperial College London, United Kingdom; d Section for Nutrition Research, Division of Digestive Diseases, Department of Metabolism, Digestion and Reproduction, Faculty of Medicine, Imperial College London, United Kingdom

**Keywords:** Mediterranean diet, Epigenetic ageing, DNA methylation, Smoking, Healthy ageing, Biological ageing

## Abstract

**Background:**

Epigenetic clocks are markers of biological ageing that may vary in their sensitivity to environmental and lifestyle exposures. To evaluate their utility as sensors of the human exposome, we investigated responses to two biologically relevant exposures with contrasting effects, the Mediterranean die and smoking.

**Objectives:**

We assessed the sensitivity of eleven epigenetic clocks to diet and smoking and evaluated whether Mediterranean diet adherence modifies associations between smoking and epigenetic ageing.

**Methods:**

We analysed 928 participants (mean age 41 years, 59% male) from the Airwave Health Monitoring Study. Linear regression models assessed associations of Mediterranean Diet Score (MDS), smoking status, and blood cotinine with epigenetic age acceleration (EAA). Smoking status × MDS interactions tested whether smoking-related EAA differed by Mediterranean diet adherence. Models were adjusted for demographic, socioeconomic, lifestyle, and psychological covariates.

**Results:**

Higher MDS was associated with lower EAA for GrimAge (β = −0.07 SD; 95% CI: −0.13, −0.01) and Bernabeu (β = −0.08 SD; 95% CI: −0.14, −0.02) after false discovery rate correction. Smoking was strongly associated with increased EAA, particularly for GrimAge, Bernabeu, and DunedinPACE. Among current smokers, effect sizes were greater in those with lower dietary adherence (e.g. GrimAge: 1.79 SD, 95% CI: 1.54, 2.04) compared with those with higher adherence (1.35 SD, 95% CI: 1.01, 1.68; P__interaction_ < 0.001). Similar attenuation patterns were observed for Bernabeu. Fruit, vegetable, and whole-grain intakes were the components most consistently associated with attenuation of smoking-related EAA.

**Conclusions:**

Our findings indicate that certain epigenetic clocks can capture the combined influence of harmful and protective exposures within the exposome. Rather than suggesting that diet neutralises the risks of tobacco, these results demonstrate variation in molecular ageing signals associated with both nutrition and environmental toxins, highlighting the potential value of second-generation clocks for quantifying resilience-related biological variation.

## Graphical abstract

**Figure:**
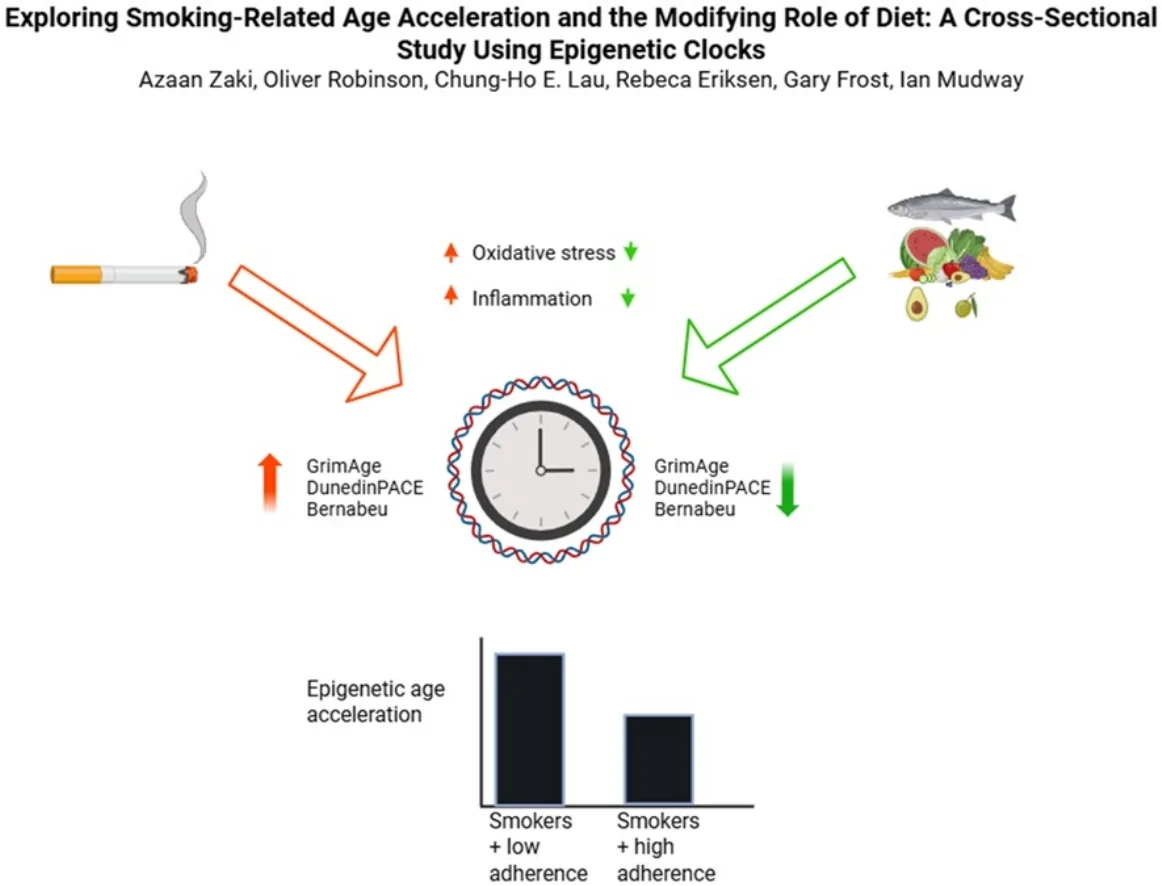

## 1 Introduction

Ageing is the most important risk factor for most chronic diseases, physical and cognitive impairment, and death [1]. Targeting ageing as a biological process may therefore represent a strategy to forestall age-associated disease [2], generating growing interest in quantifying biological age as a biomarker of chronic disease risk [3].

DNA methylation-based epigenetic clocks have been developed using different training strategies, which may influence their sensitivity to environmental and lifestyle exposures. First-generation clocks were primarily trained to predict chronological age [4,5], whereas later-generation clocks incorporate information related to physiological function, mortality risk, or longitudinal ageing processes [[6], [7], [8], [9]]. Other approaches capture specific biological processes, including telomere length and mitotic history [10,11], or incorporate genetic causal inference [12]. These later-generation measures may be particularly responsive to metabolic, inflammatory, and other biological pathways influenced by environmental and lifestyle exposures [13]. Despite the rapid expansion of epigenetic clocks, there remains a need to systematically compare their sensitivity to behavioural risk and protective factors using standardised measure [3,[14], [15], [16], [17]]. An overview of the epigenetic clocks evaluated in this study is provided in Table S1.

Diet is a key modifiable determinant of healthy ageing, yet its relationship with epigenetic ageing biomarkers remains incompletely characterised. Greater Mediterranean diet adherence has been associated with improved survival and healthier ageing trajectories [18,19], while higher overall diet quality has been associated with decelerated epigenetic ageing [20]. Mediterranean dietary components may influence ageing-related pathways through effects on metabolic health, oxidative stress and inflammation [[21], [22], [23], [24]] making this dietary pattern a useful model for investigating whether epigenetic clocks reflect variation associated with protective nutritional exposures.

In contrast, cigarette smoking is a well-characterised risk factor for premature mortality and chronic disease [[25], [26], [27], [28], [29], [30], [31], [32]], whilst increasing oxidative stress and inflammation [33], and promotes telomere attrition [34]. Smoking cessation substantially improves health outcomes [35,36], although evidence for reversal of epigenetic ageing itself remains limited. Smoking therefore provides a high-contrast environmental stressor for evaluating whether epigenetic clocks capture the biological tension between harmful exposures and protective lifestyle factors.

We examined associations of smoking and Mediterranean diet adherence with epigenetic age acceleration across eleven clocks in the Airwave Health Monitoring Study, an occupational cohort of British police employees [37]. Mediterranean diet adherence was assessed using a validated 7-day estimated-weight food diary, while smoking was characterised using self-reported smoking status, time since quitting, and blood cotinine. We hypothesised that smoking-related age acceleration would be most evident in later-generation clocks and that greater Mediterranean diet adherence would be associated with lower age acceleration and attenuation of smoking-related ageing signals.

## 2 Methods

### 2.1 Study population

This analysis used data from the Airwave Health Monitoring Study, an occupational cohort of ∼51,000 police employees in Great Britain. Full recruitment and data collection procedures are described elsewhere [37]. DNA methylation profiling was conducted on a subset of 1129 participants, selected based on the availability of high-quality DNA, genotyping, and metabolomics data. Quality control exclusions were applied for bisulfite conversion failure, low probe call rate, and gender mismatch, yielding 1115 participants with high-quality methylation data. These were subsequently merged with dietary questionnaire data collected between 2007 and 2012. In total, 928 participants had complete data for both methylation and diet variables and were included in the final analysis. Details of the study design and analyses is referred to in Fig. S1.

All participants provided written informed consent, and the study received ethical approval from the NHS Multi-Site Research Ethics Committee (MREC/13/NW/0588).

### 2.2 Dietary assessment

Dietary intake was evaluated using a 7-day estimated-weight food diary. Participants were provided with instructions, including portion size guidance based on food portion photographs and common household measurements, alongside a general questionnaire to validate the recorded intake. This diary method had been previously validated in a larger UK epidemiological study [38]. Participants submitted their completed diaries either by post or during their health screening visit at the clinic. The data were analysed using Dietplan (version 6.0; Forestfield Software), which incorporates the UK nutrient database based on *McCance and Widdowson’s The Composition of Foods* (2008, 6th edition, published by the UK Food Standards Agency). A study-specific operational manual and quality control protocol were developed to standardize the coding process and ensure the accuracy of the food diary data [39]. To evaluate potential bias due to misreporting of dietary intake, energy reporting plausibility was assessed using the Goldberg method. Reported energy intake was compared with estimated basal metabolic rate (BMR), calculated using Schofield equations based on age, sex, and body weight [40]. Cut-offs for plausible reporting incorporated within-person variation in energy intake, error in BMR estimation, and variation in physical activity level, assuming a 7-day dietary assessment period. Participants were classified as under-reporters or plausible reporters based on established criteria.

The Mediterranean Diet Score (MDS) was calculated to assess adherence to the Mediterranean dietary pattern using key dietary components Participants’ intakes of healthy food groups, including fruits, vegetables, legumes, fish, nuts, and whole grains, were scored as 1 if they exceeded the sex-specific median intake and 0 otherwise. Intakes of unhealthy food groups, including red processed meat and total dairy, were scored as 1 if they were below the sex-specific median and 0 if they exceeded it. Moderate alcohol consumption was scored as 1 for men consuming 10–50 g/day and women consuming 5–25 g/day, with scores of 0 assigned for intakes outside these ranges. The ratio of monounsaturated to saturated fatty acids (MUFA: SFA), a proxy for olive oil consumption, was also included, with a score of 1 assigned if it exceeded the sex-specific median and 0 otherwise. The total MDS, ranging from 0 to 10, was obtained by summing all component scores, where higher values indicate greater adherence to the Mediterranean diet [18,19].

### 2.3 Smoking exposure

Smoking measures included smoking status (*n* = 928), blood cotinine (*n* = 823), and time since quitting smoking among former smokers (*n* = 227). Smoking status was categorised as current, former, or never smoker, based on self-reported questionnaires. Among former smokers, time since quitting was obtained from the self-reported questionnaire item, “How long ago did you quit smoking?”, and was analysed as a continuous variable representing the reported duration since smoking cessation. Data on previous quit attempts, relapse history, smoking initiation age among former smokers, and cumulative lifetime smoking exposure were not available in a form that permitted reconstruction of lifetime smoking burden. Although the dataset included the usual number of cigarettes smoked per day before quitting, it did not include the corresponding smoking initiation information required to estimate smoking duration or pack-years reliably. Cotinine levels, an objective biomarker of recent nicotine exposure [41], were measured in blood samples using liquid chromatography–mass spectrometry (LC—MS), a highly sensitive and specific method for detecting nicotine metabolites, as previously described [42,43]. The assay provided relative cotinine values, which were normalised to account for batch effects or variations in sample processing. Cotinine values were used to cross-validate self-reported smoking status (Fig. S3). Concordance between biochemical and self-reported smoking categories was high across groups, supporting the validity of self-report data. Blood cotinine was measured for all 928 participants. Of these, 823 had non-zero cotinine values, while 105 self-reported never smokers had cotinine values of zero, consistent with their reported smoking status. Analysis of cotinine and epigenetic clocks was conducted only in participants with detectable cotinine levels.

### 2.4 Covariates

Socio-demographic, health and lifestyle data were collected via a self-administrated electronic questionnaire, which the participants filled in during their clinic visit. Variables used for the present study included: age, sex, BMI, smoking status, diet quality, household income, education level, alcohol consumption, anxiety levels, and physical activity. Models examining the MDS, the individual MDS component variables (coded as binary 0/1 variables according to the original MDS definition), or diet–smoking interaction terms were additionally adjusted for total energy intake. Covariates were defined as follows: Household income was categorised into three levels: low (<£38,000), medium (£38,000-£77,999), and high (>£78,000). Education level was classified into low (GCSE/O-Level/CSE or equivalent, vocational qualifications), medium (A-Levels/Highers or equivalent), and high (bachelor’s degree or postgraduate qualifications). Alcohol consumption was divided into three categories: non-drinker (no alcohol use), occasional drinker (≤14 alcohol units/week for women and ≤21 units/week for men), and habitual drinker (>14 units/week for women and >21 units/week for men) [41]. BMI was classified as normal weight (<25 kg/m²), overweight (25 ≤ BMI < 30 kg/m²), or obese (BMI ≥ 30 kg/m²) [42]. Physical activity was measured using the International Physical Activity Questionnaire short version and the metabolic equivalent minutes per week were calculated for each participant and categorised; high (at least 60 min. d of at least moderate-intensity activity), moderate (at least 30 min. d of at least moderate-intensity activity), low (no activity is reported or less then medium category) [43]. Anxiety was assessed using the anxiety subscale of the Hospital Anxiety and Depression Scale (HADS-A), with scores categorised as normal (0–7), borderline abnormal (8–10), and abnormal (11–21) [44]. Sex was self-reported by participants via questionnaire at the point of recruitment, using a binary male/female category; gender identity was not separately assessed in this cohort, and sex-specific dietary scoring thresholds (see Dietary assessment) were applied on this basis.

### 2.5 Epigenetic age assessment

DNA methylation data acquisition and preprocessing have been described in detail elsewhere [45]. Briefly, DNA was extracted from peripheral blood cells, and samples with insufficient DNA concentration (<25 ng/μl) were excluded. For methylation profiling, 500 ng of DNA per sample underwent bisulfite conversion (EZ DNA Methylation-Lightning™ Kit, Zymo Research, Orange, CA) and was hybridized to the Infinium HumanMethylationEPIC BeadChip. Preprocessing included quality control using SNP genotyping, background subtraction, dye-bias correction, and outlier removal based on BeadChip control intensities. DNA methylation levels were quantified as β-values and used in all statistical analyses.

DNAm-based epigenetic ages (Horvath, Hannum, PhenoAge, GrimAge, DNAmTL [[4], [5], [6], [7],^10^]) were calculated using the online DNAmAge calculator [46] from normalised DNAm data and crosschecked via the Bioconductor package Methylclock (version 0.5.0). The GrimAge algorithm incorporates DNAmPACKYRS, which is largely driven by methylation at smoking-associated loci such as cg05575921 in the AHRR gene. Bernabeu age [8], AdaptAge, DamAge, and CausAge [12], EpiTOC [11] and DunedinPACE [9] were calculated using published coefficients outlined from the original papers.

Age acceleration estimates were obtained from these raw epigenetic clock estimates by extracting the residuals from the regression model that regresses predicted age on chronological age – HorvathAA, HannumAA, PhenoAA, GrimAA, DNAmTLAdjAge, BernabeuAA, AdaptAA, DamAA, CausAA. All clock estimates (age acceleration, DunedinPACE & EpiTOC) were standardised by rescaling them to have a mean of 0 and a standard deviation of 1, to enable comparability across clocks. DNAmTLAdjAge was multiplied by -1 to align effect directionality with other clocks, such that higher values represent greater age acceleration.

### 2.6 Statistical analysis

Continuous predictors, including the MDS, blood cotinine levels, and time since quitting (in years), were also standardised (mean = 0, SD = 1) to allow interpretation of associations per SD increase in the exposure variable.

Associations between MDS and smoking measures and each epigenetic clock were assessed using linear regression models for each exposure with EAA (or corresponding clock output) as the outcome variable, adjusting for key covariates selected based on a constructed Directed Acyclic Graph (DAG) (Fig. S2) derived from the literature. These include age, sex, BMI, household income, education level, alcohol consumption, physical activity, and anxiety levels. MDS was additionally included as a covariate for analyses with smoking exposures, while smoking status was included as a covariate for analyses with MDS. Total energy intake was additionally included in models where MDS, individual dietary components, or diet–smoking interaction terms were examined.

To evaluate whether diet quality modified the association between smoking and EAA, participants within each smoking status group were divided into high and low Mediterranean diet adherence groups based on the cohort-wide median value, and differences in EAA were examined within each stratum. Next, we included an interaction term between smoking status (categorical: never, former, current) and MDS (continuous) in fully adjusted linear regression models to formally test whether the association between smoking and EAA varied by diet adherence. Interaction analyses were prioritised for epigenetic clocks demonstrating the consistent associations across smoking exposures, as effect modification is most interpretable in the presence of a clear exposure–outcome relationship. Finally, to explore which dietary components were driving any observed interaction effects, we conducted additional interaction analyses between smoking status and individual components of the MDS (e.g., fruit, vegetables, fish, whole grains, dairy), tested separately for each component, and evaluated their moderating effect on smoking-related EAA. Individual dietary components were entered as the binary Mediterranean Diet Score component variables (0/1), coded according to the original MDS definition using sex-specific median intake thresholds.

In sensitivity analysis, associations between Goldberg-defined reporting status (under-reporter vs. plausible reporter) and selected epigenetic ageing measures (GrimAge, BernabeuAA and DunedinPACE) were evaluated using linear regression models adjusted for age, sex and BMI.

Multiple testing correction was conducted using Benjamini–Hochberg False Discovery Rate FDR [47]. All analyses were performed in R version 4.3.1.

## 3 Results

### 3.1 Study population

Table 1 shows the descriptive characteristics of the subset of 928 study participants with available DNA methylation data from the Airwave cohort. The cohort’s average age is 41.0 years (SD = 9.3), and BMI is 27.2 kg/m² (SD = 4.4), with 96.9% being White and 41.2% female. These characteristics closely align with the full AIRWAVE Health and Monitoring Study cohort [37]. Education level, alcohol consumption, and anxiety significantly differed across smoking groups (*p* < 0.001, *p* = 0.006, *p* = 0.003, respectively). A higher percentage of never smokers (30.5%) had a high education level compared to smokers (15.3%) and former smokers (22.9%). Current smokers had the highest percentage of habitual drinkers (49.0%), and the highest proportion of those with abnormal anxiety levels (14.3%). Relative cotinine levels were significantly higher in current smokers compared to former and never smokers (*p* < 0.001) (Fig. S3). The MDS was significantly lower in current smokers (mean = 4.3) compared to former smokers (mean = 4.7) and never smokers (mean = 4.9), with *p* = 0.002 (Fig. S4). Based on the Goldberg method [48], 44.7% of participants were classified as dietary under-reporters and 55.3% as plausible reporters, with no substantial over-reporting observed.

**Table 1:** Baseline descriptive characteristics of AIRWAVE cohort stratified by smoking status (*N* = 928).

Characteristic		Current Smoker (*N* = 98)	Former Smoker (*N* = 227)	Never Smoker (*N* = 603)	Total (*N* = 928)	p-value
Age (Y)	Mean (SD)	42.0 (10.0)	43.4 (9.2)	40.0 (9.0)	41.0 (9.3)	<0.001
Sex	Female %	38 (38.8)	103 (45.4)	242 (40.1)	383 (41.2)	0.431
Ethnicity	White %	91 (92.9)	216 (95.2)	592 (98.2)	899 (96.9)	0.004
Relative blood cotinine (arbitrary units)	Mean (SD)	1.47 (1.12)	0.005 (0.03)	0.001 (0.00)	0.16 (0.58)	<0.001
Household Income	High %	9 (9.2)	20 (8.8)	66 (10.9)	95 (10.2)	0.794
	Medium %	57 (58.2)	143 (63.0)	369 (61.2)	569 (61.3)	
	Low %	32 (32.7)	64 (28.2)	168 (27.9)	264 (28.4)	
Education level	High	15 (15.3)	52 (22.9)	184 (30.5)	251 (27.1)	<0.001
	Medium	29 (29.6)	66 (29.1)	191 (31.7)	286 (30.8)	
	Low	54 (55.1)	109 (48.0)	228 (37.8)	391 (42.1)	
Alcohol Consumption	Habitual Drinker %	48 (49.0)	92 (40.5)	118 (19.6)	258 (27.8)	0.006
	Occasional Drinker %	38 (38.8)	108 (47.6)	192 (31.8)	338 (36.4)	
	Never Drinker %	12 (12.2)	27 (11.9)	305 (50.6)	344 (37.1)	
BMI	Mean (SD)	27.0 (3.7)	27.7 (4.9)	27.0 (17.6)	27.2 (4.4)	0.108
Physical Activity	High %	12 (12.2)	15 (6.6)	50 (8.3)	77 (8.3)	0.148
	Moderate %	10 (8.6)	13 (5.7)	47 (7.8)	70 (7.5)	
	Low %	76 (77.6)	199 (87.7)	506 (83.9)	781 (84.2)	
Anxiety	Abnormal %	14 (14.3)	22 (9.7)	40 (6.6)	76 (8.2)	0.003
	Borderline Abnormal %	10 (10.2)	45 (19.8)	92 (15.3)	147 (15.8)	
	Normal %	74 (75.5)	160 (70.5)	471 (78.1)	705 (76.0)	
Med Diet score	Mean (SD)	4.3 (1.8)	4.7 (1.8)	4.9 (1.8)	4.8 (1.8)	0.002
Q1 (Lowest)		54 (55.1)	109 (48.0)	254 (42.1)	417 (44.9)	
Q2		16 (16.3)	41 (18.1)	129 (21.4)	186 (20.0)	
Q3		14 (14.3)	39 (17.2)	97 (16.1)	150 (16.2)	
Q4 (Highest)		14 (14.3)	38 (16.7)	123 (20.4)	175 (18.9)	

Variable categorization.

Household income - Low: Income less than 38,000, Medium: Income between 38,000 and 77,999, High: Income more than 78,000.

Education level - Low: GSCE/O-Level/CSS, left school before taking O levels/GCSEs, Vocational qualifications (NVQ1 + 2). Medium: A levels/Highers or equivalent (NVQ3). High Education Level: Bachelor’s degree or equivalent (NVQ4), Postgraduate qualifications.

*Alcohol Consumption - non-drinker - No alcohol use, occasional drinker: (≤14 alcohol units/week for women and ≤21 alcohol units/week for men) or habitual drinker (>14 alcohol units/week for women and >21 alcohol units/week for men).

Physical Activity - Low: Does not meet criteria for moderate or high activity, Moderate - 5 or more days of moderate-intensity activity or walking of at least 30 min per day. High: 7 or more days of any combination of walking, moderate-intensity, or vigorous-intensity activities achieving a minimum of at least 3000 MET-minutes/week. Scoring protocol followed the International Physical Activity Questionnaire (https://sites.google.com/view/ipaq/score).

Anxiety - Probable anxiety was measured using the anxiety subscale of the HADS-A. Normal: 0–7 Borderline abnormal (borderline case): 8–10 Abnormal (case).11–21.

Values are presented as mean (SD) for continuous variables and n (%) for categorical variables. P-values compare current, former, and never smokers and were calculated using one-way analysis of variance (ANOVA) for continuous variables and Pearson's chi-square tests for categorical variables.

### 3.2 Epigenetic clock performance

The performance of each epigenetic clock in relation to chronological age, including correlation coefficients, is presented in the Supplement (Fig. S5). Horvath and Hannum had strong positive correlations with chronological age (ρ = 0.91 and ρ = 0.92, respectively; both *p* < 0.001). DNAmTL showed a moderate negative correlation (ρ = −0.52, *p* < 0.001). PhenoAge and GrimAge exhibited strong positive correlations (ρ = 0.91, *p* < 0.001; ρ = 0.89, *p* < 0.001) with chronological age in the AIRWAVE cohort. DunedinPACE and EpiTOC had weak positive correlations (ρ = 0.26, *p* < 0.001; ρ = 0.34, *p* < 0.001). Bernabeu showed a strong positive correlation (ρ = 0.90, *p* < 0.001). Moderate positive correlations were found for AdaptAge (ρ = 0.31, *p* < 0.001), while DamAge and CausAge showed strong positive correlations (ρ = 0.72, *p* < 0.001; ρ = 0.85, *p* < 0.001).

### 3.3 Mediterranean diet and EAA

**Fig. 1:**
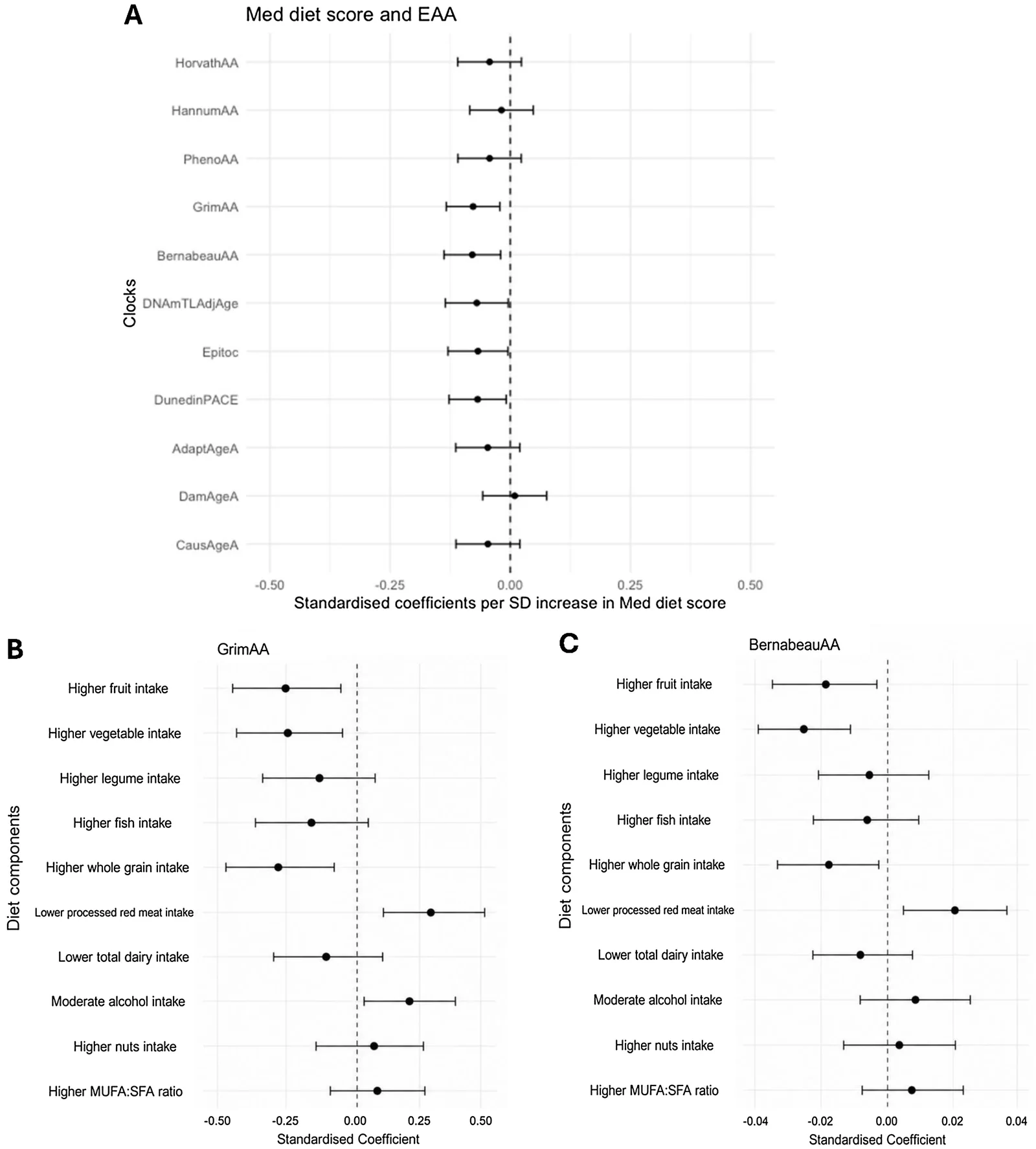
(**A**) Association between Mediterranean Diet Score (MDS) and EAA across 11 epigenetic clocks in 928 participants from the Airwave Health Monitoring Study. Effect estimates represent standardised beta coefficients per 1 SD increase in MDS, with 95% confidence intervals. Negative coefficients indicate lower EAA with greater adherence to the Mediterranean diet. (**B–C**) Associations between the individual Mediterranean Diet Score (MDS) component variables and EAA as measured by GrimAge (**B**) and BernabeuAA (**C**). Individual dietary components were entered as binary MDS variables (0/1), coded according to the original MDS definition using sex-specific median intake thresholds. Effect estimates represent standardised beta coefficients with 95% confidence intervals.

The analysis revealed inverse associations between MDS and EAA for a subset of clocks (Fig. 1A, Table S2). Each standard deviation increase in MDS was significantly associated with lower EAA as measured by GrimAge (–0.07 SD, 95% CI: –0.13, –0.01, FDR *p* = 0.033) and BernabeuAA (–0.08 SD, 95% CI: –0.14, –0.02, FDR *p* = 0.033). Nominal inverse associations were also observed for EpiTOC (–0.07 SD, 95% CI: –0.13, 0.00), DunedinPACE (–0.07 SD, 95% CI: –0.13, –0.01), and DNAmTLAdjAge (–0.06 SD, 95% CI: –0.13, 0.00), but these did not remain significant after multiple testing correction (all FDR-adjusted p-values > 0.05). No clear associations were observed for the Horvath, Hannum, PhenoAge, or causality-enriched clocks.

To identify dietary components contributing to these associations, we examined individual Mediterranean diet components in relation to GrimAge and BernabeuAA (Fig. 1B–C, Table S3). For GrimAge, higher fruit intake (β = −0.25 SD, 95% CI: −0.44, −0.05), vegetable intake (β = −0.24 SD, 95% CI: −0.43, −0.05), and whole grain intake (β = −0.27 SD, 95% CI: −0.46, −0.08) were associated with lower EAA, while lower processed red meat intake was associated with higher EAA (β = 0.27 SD, 95% CI: 0.07, 0.47); all remained significant following FDR correction. For BernabeuAA, higher vegetable intake was associated with lower EAA (β = −0.02 SD, 95% CI: −0.039, −0.011; FDR *p* = 0.005), whereas inverse associations for fruit and whole grain intake did not remain significant after multiple-testing correction.

### 3.4 Smoking and EAA

**Fig. 2:**
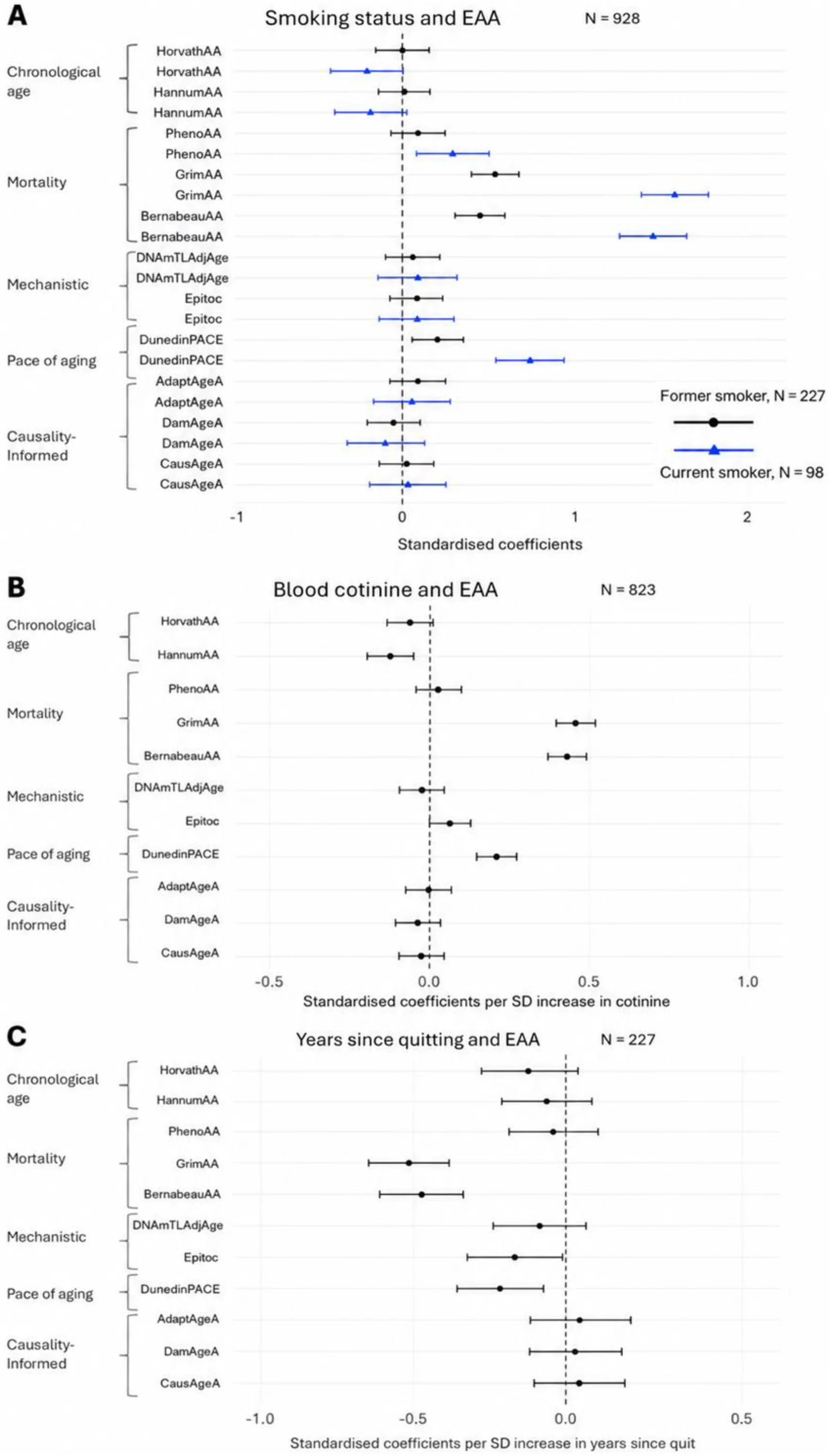
Fully adjusted regression models examining the association between smoking-related exposures and Epigenetic age acceleration (EAA). **A**: Comparison of current (*n* = 98) and former smokers (*n* = 227) to never smokers (ref: *n* = 603). **B**: Association between blood cotinine levels and EAA (*n* = 823) and **C**: Association between time since quitting smoking (in years) and EAA among former smokers (*n* = 227). Models were adjusted for age, sex, BMI, household income, education level, alcohol intake, MDS physical activity, and anxiety.

In fully adjusted models (Fig. 2A, Table S4), current smokers had higher epigenetic age acceleration compared to never smokers for PhenoAge (0.27 SD; 95% CI: 0.06, 0.49; FDR *p* = 0.043), GrimAge (1.56 SD; 95% CI: 1.37, 1.74; FDR *p* < 0.001), BernabeuAA (1.41 SD; 95% CI: 1.22, 1.61; FDR *p* < 0.001), and DunedinPACE (0.69 SD; 95% CI: 0.50, 0.89; FDR *p* < 0.001). Among former smokers, higher EAA compared to never smokers was observed for GrimAge (0.50 SD; 95% CI: 0.37, 0.64; FDR *p* < 0.001), BernabeuAA (0.42 SD; 95% CI: 0.28, 0.56; FDR *p* < 0.001), and DunedinPACE (0.19 SD; 95% CI: 0.05, 0.33; FDR *p* = 0.025).

In analyses using blood cotinine levels (Fig. 2B, Table S4), higher cotinine was associated with higher EAA for GrimAge (0.46 SD; 95% CI: 0.40, 0.52; FDR *p* < 0.001), BernabeuAA (0.43 SD; 95% CI: 0.37, 0.49; FDR *p* < 0.001), and DunedinPACE (0.21 SD; 95% CI: 0.15, 0.27; FDR *p* < 0.001). An inverse association was observed for HannumAA (–0.11 SD; 95% CI: –0.18, –0.04; FDR *p* = 0.004). The association with EpiTOC (0.06 SD; 95% CI: –0.00, 0.13) did not remain statistically significant after correction (FDR *p* = 0.120).

Among former smokers (Fig. 2C, Table S4), time since quitting was inversely associated with EAA for GrimAge (–0.50 SD; 95% CI: –0.63, –0.37; FDR *p* < 0.001), BernabeuAA (–0.46 SD; 95% CI: –0.60, –0.33; FDR *p* < 0.001), and DunedinPACE (–0.20 SD; 95% CI: –0.34, –0.07; FDR *p* = 0.012). The association with EpiTOC (–0.16 SD; 95% CI: –0.31, –0.01) did not remain significant after correction (FDR *p* = 0.099). No consistent associations were observed for the first-generation clocks (HorvathAA, HannumAA), DNAmTLAdjAge, or the causality-enriched clocks (AdaptAA, DamAA, CausAA) across smoking exposures.

### 3.5 Modification of effect of smoking on EAA, by diet

**Fig. 3:**
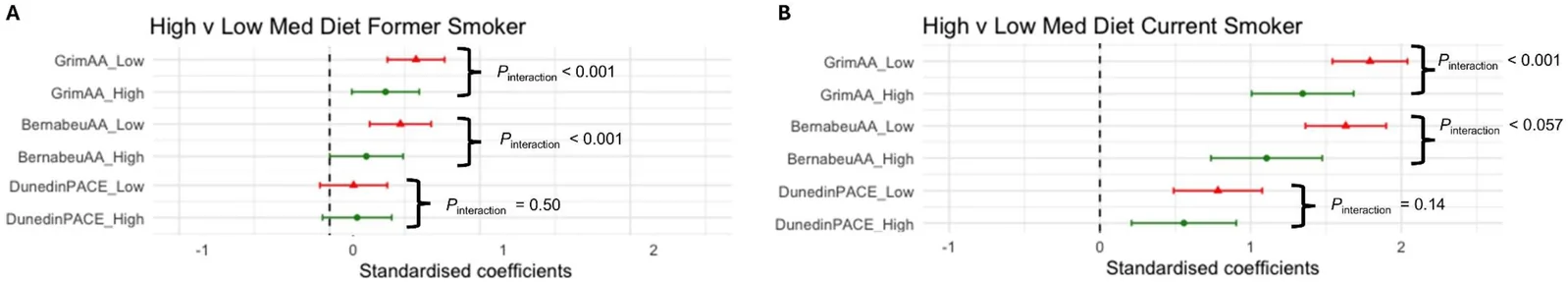
Standardised coefficients (β) with 95% confidence intervals from fully adjusted linear regression models showing the association between smoking status and epigenetic age acceleration (EAA), stratified by Mediterranean Diet Score (MDS; high vs. low, defined by cohort median). (**A**) Former vs. never smokers (*n* = 227 former smokers). (**B**) Current vs. never smokers (*n* = 98 current smokers). Models were adjusted for age, sex, BMI, household income, education, alcohol intake, physical activity, and anxiety. P_interaction values were derived from models including a continuous MDS × smoking status interaction term and are shown for selected clocks.

Stratified analyses by high vs. low MDS revealed differences in associations between smoking status and EAA across subgroups (Fig. 3 Table S5). Among former smokers, GrimAge and Bernabeu remained significantly associated with elevated EAA across both adherence groups but were attenuated with higher adherence. GrimAge showed a 0.57 SD increase in the low group (95% CI: 0.38, 0.77), compared to 0.37 SD in the high group (95% CI: 0.15, 0.60). Bernabeu was 0.47 SD (95% CI: 0.27, 0.68) in the low group vs. 0.24 SD (95% CI: 0.00, 0.49) in the high group. DunedinPACE estimates were similar across diet strata among former smokers: 0.19 SD (95% CI: −0.04, 0.41) in the low adherence group and 0.19 SD (95% CI: −0.04, 0.43) in the high adherence group. Interaction terms were statistically significant for GrimAge and BernabeuAA (both *P*_interaction_ < 0.001), but not for DunedinPACE (*P*_interaction_ = 0.50). Among current smokers, those with low diet adherence exhibited greater EAA compared to never smokers, especially for GrimAge (1.79 SD, 95% CI: 1.54, 2.04) and Bernabeu (1.63 SD, 95% CI: 1.36, 1.90). These effects were attenuated in the high adherence group, with GrimAge at 1.35 SD (95% CI: 1.01, 1.68) and Bernabeu at 1.11 SD (95% CI: 0.74, 1.48). Interaction terms confirmed statistically significant moderation by MDS for GrimAge (P_interaction_ < 0.001), whereas evidence for interaction with BernabeuAA was weaker and did not reach statistical significance (*P*_interaction_ = 0.057). For DunedinPACE, effect estimates were also higher among individuals with lower diet adherence (0.78 SD; 95% CI: 0.49, 1.08) compared with those with higher adherence (0.56 SD; 95% CI: 0.21, 0.90); however, no interaction was observed for DunedinPACE (P_interaction_ = 0.14).

**Fig. 4:**
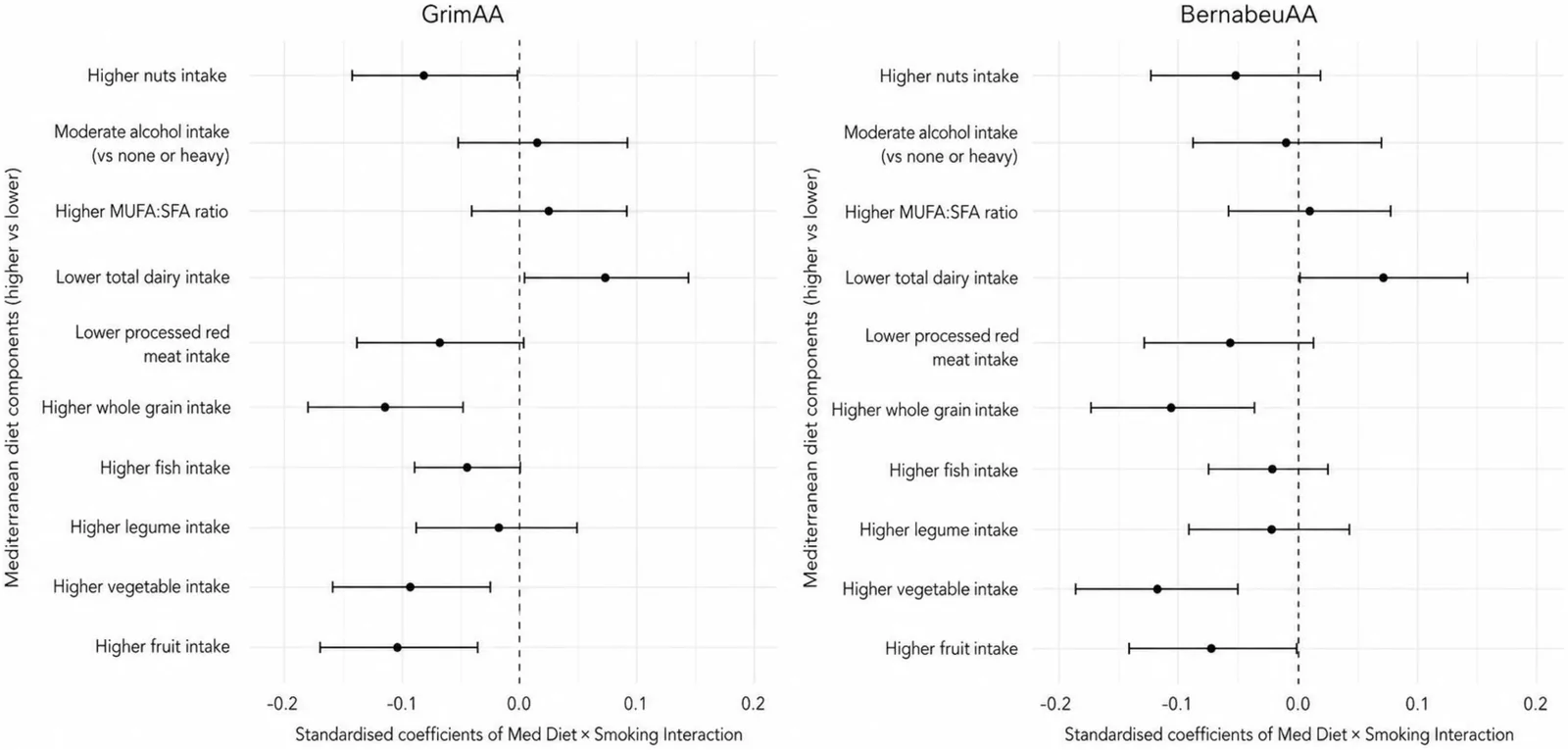
Standardised coefficients (with 95% CIs) from fully adjusted regression models showing the interaction effects between smoking status and individual Mediterranean Diet components on EAA, for GrimAge and BernabeuAA. Each point represents the standardised coefficient for the interaction term (Smoking × Diet component), indicating how dietary intake modifies the association between smoking and EAA. Negative values suggest that higher intake of the component attenuates smoking-related age acceleration. Models were adjusted for age, sex, BMI, household income, education level, alcohol intake, physical activity, anxiety and total energy intake.

Further interaction analyses of individual Mediterranean diet components showed that several food groups modified the association between smoking and epigenetic age acceleration, particularly for GrimAge and BernabeuAA (Fig. 4, Table S6). For GrimAge, interactions terms for higher fruit intake (−0.12 SD, 95% CI: −0.19, −0.05), vegetables (−0.08 SD, 95% CI: −0.15, −0.01), whole grains (−0.12 SD, 95% CI: −0.19, −0.05), and lower dairy intake (0.09 SD, 95% CI: 0.02, 0.16) were significant after correction for multiple testing. For BernabeuAA, significant interactions were observed for higher vegetable intake (−0.12 SD, 95% CI: −0.19, −0.05) and higher whole grain intake (−0.11 SD, 95% CI: −0.18, −0.04). Associations for other components, including fruit and fish intake, were attenuated after correction for multiple testing.

### 3.6 Sensitivity analysis

Sensitivity analyses comparing dietary under-reporters with plausible reporters showed no significant differences in GrimAge, BernabeuAA or DunedinPACE after adjustment for age, sex and BMI (Table S7).

## 4 Discussion

This study demonstrates that later-generation epigenetic clocks effectively capture the biological tension between harmful environmental stressors and protective lifestyle factors. We found that higher adherence to a Mediterranean diet was associated with significantly lower epigenetic age acceleration. This relationship was particularly second-generation mortality-trained clocks such as Bernabeu and GrimAge. By using smoking as a high-contrast benchmark, we further established that these specific clocks could detect the potential for dietary patterns to attenuate the molecular damage associated with tobacco smoke exposure.

Our findings are consistent with growing evidence that higher Mediterranean diet adherence is associated with slower epigenetic ageing. For example, our results align with those of Kim and colleagues [20], who reported inverse associations between Mediterranean diet scores and both GrimAge and the precursor to DunedinPACE. They are also supported by recent findings from the Women's Health Initiative, in which higher adherence to Healthy Eating Index, DASH, and alternative Mediterranean dietary patterns was associated with lower epigenetic age acceleration, particularly for GrimAge, PhenoAge, and DunedinPACE, across 4500 postmenopausal women [49]. Consistent with our results, associations were strongest for second-generation ageing biomarkers and measures of pace of ageing. We observed comparable effect sizes of approximately –0.08 standard deviations per 1 standard deviation increase in diet score. Further supporting evidence from the NU-AGE intervention study showed that a one-year Mediterranean-style dietary intervention was associated with lower Horvath-derived epigenetic age acceleration measures [50]. We extend these findings to additional clocks, including Bernabeu, EpiTOC, and DNAmTL, although we did not replicate the PhenoAge association reported in previous studies.

The strong associations observed for smoking provide an internal benchmark for interpreting these findings. Current smokers showed pronounced age acceleration relative to never smokers, with GrimAge showing the strongest response. Prior studies have associated smoking with a reduction in life expectancy of approximately 10 years [32]. Given that a 1-year GrimAge acceleration approximately corresponds to a hazard ratio of 1.10 for mortality [7], the 7.18-year acceleration observed among current smokers is consistent with a marked increase in mortality risk and aligns with evidence of substantial life expectancy reductions among smokers [51]. This finding is expected, given that GrimAge, like Bernabeu, incorporates smoking history into its training algorithm. PhenoAge is driven by a limited set of broad systemic markers, including inflammation and metabolic dysfunction; consequently, smoking-related signals may be less pronounced, particularly among individuals with healthier baseline physiology or among former smokers. Although PhenoAge captures long-term ageing risk, it appeared less sensitive to smoking in this cohort. In contrast, DunedinPACE more clearly captured smoking-related ageing. DunedinPACE incorporates markers across multiple systems, including cardiovascular function (e.g., mean arterial pressure), pulmonary function (FEV1), metabolic health (BMI, HbA1c), and immune activity (CRP), all of which are affected by tobacco exposure [9]. Our finding that GrimAge and DunedinPACE were most sensitive to smoking mirrors the ranking reported by Klopack et al. [52], who assessed lifetime smoking exposure, reinforcing the relative sensitivity of these clocks to tobacco exposure.

Blood cotinine levels further reinforced these trends, showing dose-dependent associations with GrimAge, Bernabeu, and DunedinPACE. EpiTOC, a mitotic clock capturing cell division rates, showed a borderline association with cotinine, suggesting that smoking-induced replication stress may contribute to accelerated ageing. Additionally, our blood-based analysis showed that longer time since smoking cessation was associated with reduced epigenetic age acceleration as measured by GrimAge, Bernabeu, and DunedinPACE, with a nominal association observed for EpiTOC. However, these findings should be interpreted cautiously because time since quitting captures only one aspect of smoking history. Repeated quit attempts, relapse patterns, and the timing of smoking cessation may reflect both smoking-related biological responses and broader health or lifestyle characteristics. In addition, smoking cessation may occur for health-related reasons, including emerging age-related disease, which could introduce reverse causation or healthy-quitter effects. Because the available data did not capture smoking initiation age, relapse history, number of quit attempts, cumulative pack-years among former smokers, or health-related reasons for cessation, we could not formally evaluate these mechanisms or distinguish the effects of abstinence duration from prior smoking burden. We therefore avoid attributing the time-since-quitting findings solely to duration of abstinence. Future studies with detailed longitudinal smoking histories are needed to disentangle these effects. Our models were nevertheless adjusted for several potential lifestyle and socioeconomic confounders, including Mediterranean diet adherence, physical activity, BMI, alcohol consumption, education, and household income. On the contrary, some clocks, particularly the first-generation Horvath and Hannum clocks, as well as the causality-enriched clocks (AdaptAge, CausAge, and DamAge), did not demonstrate significant associations with smoking indices. Horvath and Hannum clocks, designed to predict chronological age, showed no significant associations with smoking, suggesting their reliance on time-dependent CpG changes rather than those influenced by external stressors.

Given the consistent diet associations across clocks, we examined whether diet modified susceptibility to smoking-related age acceleration. For the clocks most strongly associated with smoking, including DunedinPACE, GrimAge, and Bernabeu, associations with smoking status were generally stronger among participants with low Mediterranean diet adherence. Because these clocks partly reflect methylation profiles associated with smoking, this observation cannot be interpreted as evidence of a direct effect on biological ageing. It does, however, suggest that smoking-related mortality-associated epigenetic signals are smaller among individuals with higher adherence to a nutritious dietary pattern. This interaction was most evident for GrimAge, for which the antioxidant and anti-inflammatory features of a Mediterranean dietary pattern may be relevant to oxidative and inflammatory pathways activated by tobacco exposure. Component-level analyses indicated that higher intakes of fruits, vegetables, and whole grains were most consistently associated with attenuation of smoking-related EAA, consistent with broader evidence linking these dietary patterns to reduced systemic inflammation and oxidative stress [53,54]. These findings closely mirror those reported by Kim et al. [20], who observed inverse associations between fruit, vegetable, whole grain, and legume intake and GrimAge acceleration. The consistency of these component-level associations across cohorts suggests that specific features of high-quality dietary patterns may be important determinants of biological ageing signals. Notably, Kim et al. also reported stronger associations between dietary components and epigenetic ageing among ever-smokers than never-smokers, which is consistent with our observation that dietary patterns appear most informative in the context of smoking-related ageing signals.

The observation that fibre-rich dietary components were associated with attenuation of smoking-related age acceleration is supported by a recent study showing that higher fibre intake was associated with greater odds of healthy ageing in a large cohort [24]. These findings are also biologically plausible given experimental evidence that a polyphenol-rich Mediterranean dietary intervention can induce widespread changes in DNA methylation and gene expression, particularly in pathways related to inflammation and one-carbon metabolism [55]. These lines of evidence suggest that a healthy diet may buffer the biological impact of environmental pollutants more generally. Such results may be applicable to other oxidative stressors, such as ambient air pollution, which operate through similar pathways of inflammation and metabolic disruption.

Together, these lines of evidence suggest that a healthy diet may modify biological responses to environmental stressors more broadly. This may be relevant to other oxidative stressors, such as ambient air pollution, which operate through overlapping pathways of inflammation and metabolic disruption.

### 4.1 Strengths and limitations

This study provides a comprehensive evaluation of the sensitivity of epigenetic clocks to smoking and of the potential moderating influence of diet. Midlife is a pivotal period in which lifestyle exposures may exert measurable effects on biological ageing. In populations where extensive longitudinal health data or overt disease may be absent, epigenetic clocks offer a useful approach for quantifying biological impact. The large cohort size and detailed smoking measures, including both self-reported status and blood cotinine, strengthen the robustness of our findings. In addition, seven-day food diaries provide a more detailed assessment of dietary intake than food frequency questionnaires or 24-h recalls, enabling more granular analysis of specific Mediterranean diet components.

Several limitations should be considered. The cross-sectional design limits causal inference, and the predominantly white occupational cohort may restrict generalisability. A key limitation is the distribution of smoking status, with approximately six times more non-smokers than smokers. This imbalance reflects both the contemporary decline in smoking prevalence and the occupational nature of the cohort, but it may also introduce a healthy worker effect and lead to underestimation of smoking-related age acceleration. The relatively small number of current smokers with high Mediterranean diet adherence also reduces statistical power, so results in these subgroups should be interpreted cautiously. In addition, participants who completed food diaries may differ from those who did not, introducing potential selection bias. A proportion of participants were classified as under-reporters using the Goldberg method; however, sensitivity analyses indicated that dietary misreporting is unlikely to materially explain the observed associations. Models examining Mediterranean diet exposures were also adjusted for total energy intake, reducing the potential impact of systematic under-reporting. Future studies should incorporate objective dietary validation methods, such as metabolomic profiling or measurement of specific nutrients, to minimise self-reporting bias.

## 5 Conclusion

In conclusion, this study provides a comparative evaluation of diverse epigenetic ageing biomarkers by examining their responsiveness to healthy diet, smoking, and their interaction as protective and hazardous components of the exposome. The associations of specific dietary components, particularly fruits, vegetables, and whole grains, with smoking-related age acceleration reinforce the biological plausibility of these clocks. Specifically, attenuation of age acceleration in clocks incorporating smoking-related scores suggests that methylation changes are not merely static markers of exposure, but may also reflect dynamic biological processes associated with lifestyle factors. These findings support the value of second-generation clocks for capturing biologically meaningful and potentially modifiable aspects of ageing. They also suggest that the increased risk of age-related disease associated with smoking may be exacerbated by the absence of a healthy diet, underscoring the potential relevance of dietary patterns to biological resilience.

## CRediT authorship contribution statement

Conceptualization: I.M., O.R., A.Z. Methodology: A.Z., I.M., O.R. Formal analysis: A.Z. Investigation: C.L. Data curation: C.L. Writing - original draft: A.Z. Writing - review & editing: I.M., O.R., C.L., R.E., G.F., A.Z. Supervision: I.M., O.R., C.L. All authors critically reviewed and approved the final manuscript.

## Ethical statement

Ethical approval was obtained from the National Health Service Multi‐Site Research Ethics Committee (MREC/13/NW/0588). All participants provided written informed consent.

## Declaration of Generative AI and AI-assisted technologies in the writing process

During the preparation of this work, the authors used generative AI-assisted technologies to improve language and readability. After using these tools, the authors reviewed and edited the content as needed and take full responsibility for the content of the publication.

The authors did not use generative AI or AI-assisted technologies in the writing of this manuscript or in the preparation of figures, images, or artwork.

## Funding

This work was supported by the Medical Research Council Centre for Environment and Health (MR/S019669/1) and UKRI Dream (NE/T001895/1). The Airwave Health Monitoring Study was funded by the 10.13039/501100000824Home Office (grant number 780-TETRA), with additional support from the 10.13039/501100000272National Institute for Health Research (NIHR) Biomedical Research Centre. Computing resources were provided by the UK MEDical BIOinformatics Partnership (UK MED-BIO), supported by the 10.13039/501100007155Medical Research Council (MR/L01632X/1). O.R. and C.H.L. were supported by a UKRI Future Leaders Fellowship (MR/S03532X/1, MR/Y02012X/1).

## Data availability

This research has been conducted with the AIRWAVE Health and Monitoring Study. Data are requestable through https://police-health.org.uk/applying-access-resource

## Declaration of competing interest

The authors have declared no conflict of interest.
